# Comparative Proteomic Analysis of Type 2 Diabetic versus Non-Diabetic Vitreous Fluids

**DOI:** 10.3390/life14070883

**Published:** 2024-07-16

**Authors:** Abdulaziz H. Alanazi, Shengshuai Shan, S. Priya Narayanan, Payaningal R. Somanath

**Affiliations:** 1 Clinical and Experimental Therapeutics, University of Georgia, Augusta, GA 30912, USA; 2 Vision Discovery Institute, Augusta University, Augusta, GA 30912, USA; 3 Department of Clinical Practice, College of Pharmacy, Northern Border University, Rafha 91531, Saudi Arabia

**Keywords:** diabetes, vitreous, proteomics, inflammation, gene enrichment analysis

## Abstract

Background: Diabetic retinopathy (DR) is a leading cause of vision loss, with complex mechanisms. The study aimed to comprehensively explore vitreous humor of diabetic and non-diabetic individuals, paving the way for identifying the potential molecular mechanisms underlying DR. Methods: Vitreous samples from type 2 diabetic and non-diabetic subjects, collected post-mortem, were analyzed using liquid chromatography–mass spectrometry. Pathway enrichment and gene ontology analyses were conducted to identify dysregulated pathways and characterize protein functions. Results: Pathway analysis revealed dysregulation in multiple metabolic and signaling pathways associated with diabetes, including glycerolipid metabolism, histidine metabolism, and Wnt signaling. Gene ontology analysis identified proteins involved in inflammation, immune response dysregulation, and calcium signaling. Notably, proteins such as Inositol 1,4,5-trisphosphate receptor type 2 (ITPR2), Calcium homeostasis endoplasmic reticulum protein (CHERP), and Coronin-1A (CORO1A) were markedly upregulated in diabetic vitreous, implicating aberrant calcium signaling, inflammatory responses, and cytoskeletal reorganization in DR. Conclusions: Our study provides valuable insights into the intricate mechanisms underlying DR and highlights the significance of inflammation, immune dysregulation, and metabolic disturbances in disease progression. Identification of specific proteins as potential biomarkers underscores the multifactorial nature of DR. Future research in this area is vital for advancing therapeutic interventions and translating findings into clinical practice.

## 1. Introduction

Diabetic retinopathy (DR), the most common cause of vision loss in working-age adults in the US, presents a daunting challenge in both its pathology and available treatments [1]. Despite extensive research, the precise mechanisms triggering its onset and progression remain elusive. Factors such as prolonged hyperglycemia, the generation of pro-inflammatory and pro-angiogenic proteins, lipids, and metabolites, and oxidative damage contribute to the impairment of retinal blood vessels [2,3,4,5]. The lack of a comprehensive understanding of its pathology hampers the development of effective therapies. Current treatments such as vascular endothelial growth factor (VEGF) inhibitors and corticosteroids primarily target late-stage manifestations to manage macular edema and neovascularization [6]. However, these interventions often come after irreversible damage has occurred [6,7]. Novel diagnostic tools, including optical coherence tomography angiography (OCT-A), show promise in early detection, yet the challenge persists in preventing disease progression [8]. Addressing the complex pathology of DR demands concerted efforts in unraveling its molecular underpinnings to pave the way for innovative therapeutic strategies aimed at halting its advancement from the early stages.

The vitreous humor, a vital gel-like substance bathing the posterior segment of the eye, is essential for maintaining ocular structure and function [9]. Its composition reflects a delicate equilibrium of proteins, electrolytes, and growth factors, crucial for ocular health. Previous research has provided valuable insights into the dynamic nature of the vitreous composition, highlighting its role in intraocular pressure regulation, retinal support, and nutrient transport [10,11,12,13,14]. However, in the context of diabetes mellitus, a metabolic disorder characterized by hyperglycemia, the vitreous undergoes significant alterations, reflecting the pathogenesis of DR, a leading cause of vision loss globally [15,16,17]. The multifactorial nature of DR involves complex interplays of biochemical pathways, with vitreous composition playing a pivotal role [18,19].

Recent studies have elucidated the presence of inflammatory cytokines, angiogenic factors, and extracellular matrix proteins in the vitreous of diabetic individuals, underscoring their involvement in retinal damage and neovascularization [17,19]. Advances in proteomic techniques have facilitated the identification of novel biomarkers associated with DR progression [19,20]. Despite these advancements, the available data often combine results from both type 1 and type 2 diabetic patients, along with compounding factors such as diverse treatments and co-morbidities. A comprehensive understanding of vitreous composition in type 2 diabetes remains unclear, necessitating further investigation.

Comparative studies analyzing vitreous humor from diabetic and non-diabetic individuals are crucial for deciphering the molecular mechanisms underlying DR and identifying potential therapeutic targets. Therefore, in the present study, we aimed to address this gap by conducting a detailed liquid chromatography–mass spectrometry (LC–MS) proteomic analysis of vitreous humor samples sourced from both normal subjects and patients with type 2 diabetes mellitus. Through rigorous examination of protein profiles and inflammatory mediators, we endeavored to delineate the distinct biochemical signatures associated with diabetes and their implications for DR pathogenesis.

## 2. Materials and Methods

### 2.1. Source of Samples and Preparation for LC–MS

The vitreous samples and their corresponding coded information from diabetic and non-diabetic individuals (postmortem) were obtained from the National Disease Research Interchange, Philadelphia, PA, USA. The vitreous humor samples were filtered and purified from any cells or debris through a centrifugation process at 2000× *g* for 20 min at 4 °C. The BCA protein assay kit (Cat#23225, Thermo Scientific, Waltham, MA, USA) was utilized to quantify the protein concentrations (μg/μL) of the attained vitreous samples by following the manufacturer’s instructions. In total, 50 μg of total protein was prepared and subjected to LC–MS analysis (proteomics core, Augusta University).

### 2.2. Protein Identification and Data Analysis

Proteome Discoverer software (version 1.4, Thermo Scientific) processed the raw mass spectrometry data. Peptide sequences were detected against the SwissProt human database using SequestHT search with 10 ppm precursor ion tolerance and 0.6 Da production tolerance. Data transformation and normalization were performed using the Trimmed Mean of M-values (TMM) with the R library. Protein abundance changes between diabetic and non-diabetic vitreous samples were evaluated using Student’s *t*-test (*p*-value < 0.05, fold change > 2) after normalization. Peptide spectral match (PSM) counts provided a semi-quantitative measure of protein abundance. R version 4.3.2 was used for protein distribution analysis, including PCA, volcano plot, and hierarchical clustering. The Venn diagram was developed to provide an overview of the total analyzed proteins, demonstrating the number of unique proteins in each group. Principal component analysis was used to confirm that both groups were distinguished from each other. The volcano plot and clustering heatmap were designed to show the differentially expressed proteins in diabetic vitreous samples. R version 4.3.2 was used to perform the above analyses. Differentially expressed proteins underwent functional, kinase enrichment, and network analyses using Enricher and SRplot [21,22,23]. The area under the receiver operating characteristic curve (AUC) was calculated using GraphPad Prism version 9. The identified proteins were analyzed using pathway databases including KEGG, BioCarta, Panther, and Reactome. The cellular components, biological processes, and molecular functions of significantly differentially expressed proteins were explored using Gene Ontology (GO). Protein–protein interactions were analyzed using the STRING platform [24].

### 2.3. Statistical Analysis

The protein changes between vitreous samples of diabetic and non-diabetic subjects were evaluated using Student’s *t*-test (*p*-value and fold change). Proteins with fold change values > 0.5 or <0.5 are considered upregulated and downregulated, respectively. Descriptive statistics were applied to assess patient demographics using IBM SPSS Statistics Version 29. In all tests, a *p*-value less than 0.05 is deemed to be statistically significant.

## 3. Results

### 3.1. Patient Characteristics

The vitreous fluids were taken post-mortem from 12 subjects, of which 6 had diabetes, while the others were non-diabetic (Table 1). The baseline characteristics of the study subjects indicated that the average age between the diabetic and non-diabetic groups was close to being matched, 72 ± 12.17 and 79.83 ± 11.34 years old, respectively. Similarly, other baseline characteristics, including gender, race, presence of health complications such as heart attack, and the use of tobacco or alcohol, were not different. However, the analyses were not at a statistically significant level, indicating that these variables were not different between the two groups. Expectedly, the group with type 2 diabetes showed around 14 years of living with the disease compared to non-diabetic subjects.

### 3.2. Proteomic Profiling of Diabetic Vitreous Samples

**Figure 1:**
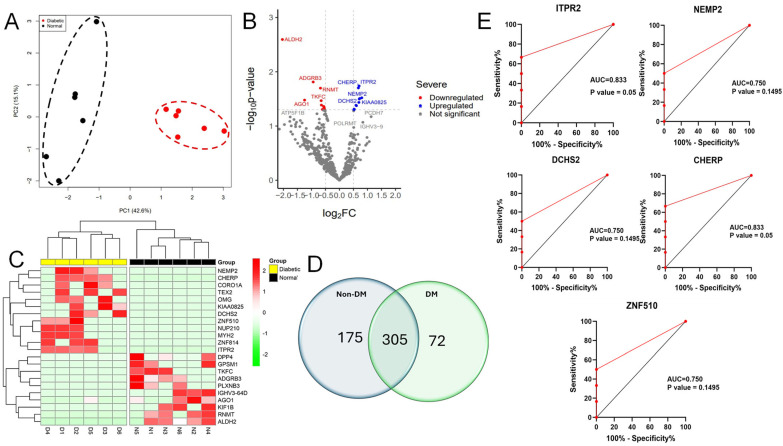
Proteins with differential expression in the diabetic vitreous. (**A**) Principal component analysis (PCA) indicating the two groups were separated. (**B**) The volcano plot of diabetic vitreous versus non-diabetic samples demonstrates the quantifiable proteins, including 22 proteins that are significantly altered among the groups. (**C**) The heatmap shows 22 differentially expressed proteins in diabetic compared to non-diabetic vitreous specimens. (**D**) Venn diagram showing the detailed counts of identified proteins. (**E**) The area under the receiver operating characteristic curve (AUC) for the top 5 significant upregulated proteins, including ITPR2, CHERP, DCHS2, ZNF510, and NEMP2.

The mass spectrometry analysis of 12 vitreous specimens revealed the presence of 4346 unique proteins in all groups. Out of the proteins analyzed, only 552 were found in at least 50% of all samples analyzed (Figure 1). Among 552 proteins, 175 were recognized in the vitreous of the non-diabetic group, 72 were only detected in the diabetic group, and 305 were common in all the samples (Figure 1D). Proteins that were abundantly expressed in the vitreous fluid samples are included in Table 2.

Despite the small sample size, we further explored the possibility of identifying a biomarker among the differentially expressed proteins. The top five significant upregulated proteins, ITPR2, CHERP, DCHS2, ZNF510, and NEMP2, were evaluated using receiver operating characteristic (ROC) curve analyses. Our analysis showed that among all the above-analyzed proteins, ITPR2 and CHERP proteins are potential biomarkers with an AUC equal to 0.8 and a *p*-value that is significant (Figure 1E).

### 3.3. A Comprehensive Gene Enrichment Analysis of the Differentially Expressed Proteins

Using principal component analysis (PCA) in our protein distribution study, we found a clear separation between the diabetic and non-diabetic groups based on their protein expression variances (Figure 1A). To investigate more deeply the difference in protein expression levels seen at significant levels between the two groups, we found 22 proteins incorporating 12 upregulated and 10 downregulated proteins in vitreous samples of diabetic subjects as demonstrated in the volcano plot and hierarchical clustering heatmap (Figure 1B,C). Proteins, namely nuclear envelope integral membrane protein 2, zinc finger protein, oligodendrocyte-myelin glycoprotein, coronin-1A, inositol 1,4,5-trisphosphate receptor type 2, protocadherin-23, calcium homeostasis endoplasmic reticulum protein, and myosin-2, were detected to be significantly increased in diabetic versus non-diabetic vitreous. In contrast, other proteins, including immunoglobulin heavy variable 3-64D, mRNA cap guanine-N7 methyltransferase, adhesion G protein-coupled receptor B3, kinesin-like protein KIF1B, aldehyde dehydrogenase, mitochondrial, dipeptidyl peptidase 4, triokinase/FMN cyclase, G-protein-signaling modulator 1, protein argonaute-1, and plexin-B3, were observed to be expressed at lower levels in diabetic samples (Table 3 and Table 4).

**Figure 2:**
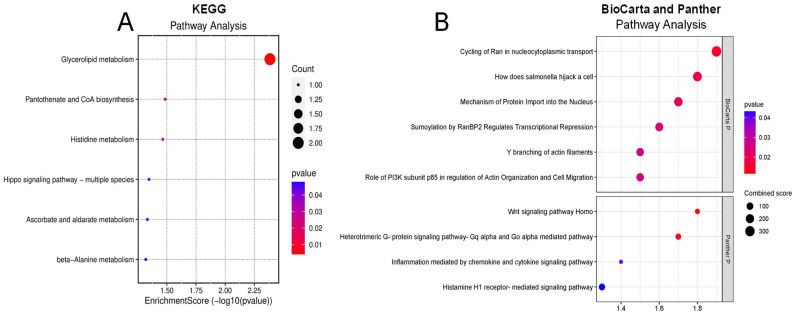
Enrichment pathway analysis of differentially expressed genes. (**A**) KEGG and (**B**) Panther (upper) and BioCarta (lower) pathway analysis of diabetic of vitreous showing potential involvement of inflammatory pathways.

To understand the potential pathways involved in retinal damage leading to DR, we analyzed significantly altered proteins in the vitreous humor of diabetic subjects using pathway enrichment analyses from Panther, KEGG, Reactome, and BioCarta databases. We identified 26 dysregulated pathways within the vitreous of diabetic patients in all the databases. The top KEGG pathways that are significantly connected to changed proteins in diabetes are glycerolipid metabolism, pantothenate and CoA biosynthesis, histidine metabolism, and ascorbate and aldarate metabolism (Figure 2A). According to the Panther database, we found several signaling pathways, including Wnt, heterotrimeric G-protein (Gαq and Gαo)-mediated pathway, inflammation mediated by chemokine and cytokine signaling pathway, and histamine H1 receptor-mediated signaling pathways were significantly involved in altered proteins in the diabetic vitreous (Figure 2B). Although the BioCarta database demonstrated a few altered pathways, the cycling of Ran in nucleocytoplasmic transport and the role of PI3K subunit p85 in the regulation of actin organization and cell migration were the most pertinent pathways to retinal pathogenesis (Figure 2B). Upon examining the markedly altered proteins in Reactome enrichment analysis, we detected pathways such as transcriptional regulation by MECP2, Ca^2+^ pathway, transcriptional regulation by small RNAs, fructose catabolism, MET/PTPN11, and MET/TNS proteins significantly dysregulated in the diabetic vitreous (Table 5).

### 3.4. Gene Ontology, Kinase Enrichment Analyses, and Protein–Protein Interactions

**Figure 3:**
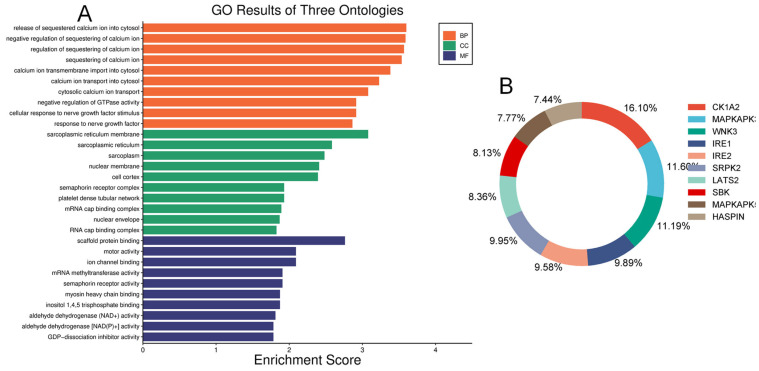
Gene ontology and Kinase enrichment analysis. (**A**) Top enriched ontologies include biological processes (BP), cellular components (CC), and molecular functions (MF) of differentially expressed proteins in diabetic specimens. (**B**) The top ten protein kinases of differentially expressed proteins in diabetic vitreous.

We examined and mapped the biological roles, molecular functions, and cellular components of differentially expressed proteins using gene ontology (GO) analysis. In the diabetic vitreous, the top molecular functions of altered proteins were associated with scaffold protein binding, motor activity, ion channel binding, myosin binding, RNA binding, dipeptidyl-peptidase activity, semaphorin receptor activity, and GDP-dissociation inhibitor activity. The enriched biological roles included positive chemotaxis, negative regulation of GTPase activity, negative regulation of calcium ion sequestration, release of sequestered calcium ions into the cytosol, response to nerve growth factor, and calcium ion transmembrane import into the cytosol. Analysis of cellular components revealed highly enriched processes related to the sarcoplasmic reticulum membrane, sarcoplasmic reticulum, cyclin/CDK positive transcription elongation factor complex, platelet dense tubular network membrane, platelet dense tubular network, cell cortex, mRNA cap-binding complex, myosin filament, and endocytic vesicle (Figure 3).

**Figure 4:**
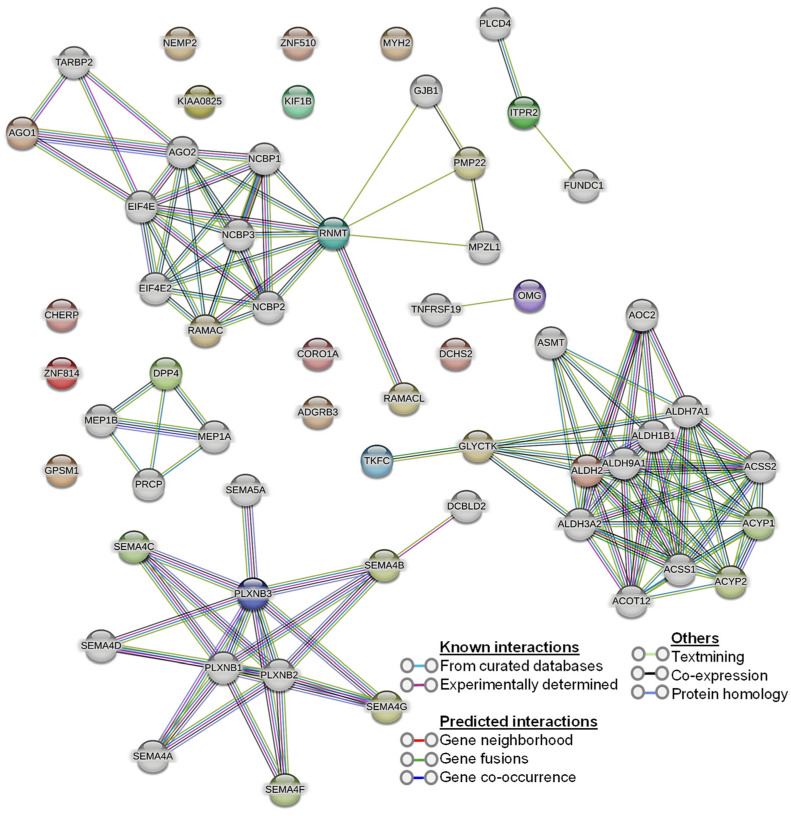
Protein–protein interaction (PPI) network of the 22 differentially expressed proteins from the STRING database.

Our human phenotype ontology analysis showed that attenuation of retinal blood vessels was significantly related to altered proteins in diabetic vitreous samples (Table 6). The STRING database was used to identify the protein−protein interactions in differentially expressed upregulated and downregulated proteins. We observed diverse networks among the proteins. However, some proteins did not show interaction, indicating that these proteins need to be investigated to clarify such connections. We also explored the significantly altered proteins in diabetic vitreous in the kinase library using Enricher. As a result, we observed numerous significantly enriched kinases incorporating casein. Kinase A 1 is the most enriched kinase, followed by MAPKAPK3/5 and WNK3 (Figure 3B). A detailed protein–protein interaction network of 22 differentially expressed proteins in diabetic vitreous from STRING database analysis is presented in Figure 4.

## 4. Discussion

DR is a leading cause of vision impairment and blindness in diabetic patients worldwide, driven by chronic hyperglycemia, inflammation, and associated metabolic disturbances [1,2]. Understanding its molecular mechanisms is crucial for developing effective therapies. Proteomic analysis of vitreous humor has emerged as a powerful tool for identifying biomarkers and elucidating the pathophysiology of DR [18,19]. Recent advancements in mass spectrometry and bioinformatics enable comprehensive profiling of the vitreous proteome, providing insights into inflammation, angiogenesis, and extracellular matrix remodeling in proliferative DR [25,26]. However, apart from smaller sample sizes and technical variability, other limitations of these studies include compounding factors from different cohorts of patient characteristics and treatments of various conditions they received. Our study focuses on proteomic analysis of vitreous samples from diabetic and non-diabetic subjects, revealing proteins significantly associated with metabolic regulation and inflammation, and highlighting alterations in pathways such as Wnt and cytokine-induced inflammation. This multifaceted approach enhances our understanding of possible pathways involved in DR development and aids in identifying novel therapeutic targets.

The comprehensive gene enrichment analysis of differentially expressed proteins in the diabetic vitreous humor versus non-diabetic vitreous revealed important insights into how diabetes affects vitreous content. Principal component analysis (PCA) demonstrated a clear separation between diabetic and non-diabetic groups based on protein expression variances, underscoring the distinct proteomic alterations associated with DR and aligning with previous studies that have reported significant changes in the vitreous proteome of DR patients [27,28,29]. We identified 22 differentially expressed proteins, with 12 upregulated and 10 downregulated in diabetic samples. Notably, proteins such as nuclear envelope integral membrane protein 2 (NEMP2), zinc finger protein 814 (ZNF814), oligodendrocyte-myelin glycoprotein (OMG), coronin-1A (CORO1A), inositol 1,4,5-trisphosphate receptor type 2 (ITPR2), protocadherin-23 (DCHS2), calcium homeostasis endoplasmic reticulum protein (CHERP), and myosin-2 (MYH2) were significantly increased in diabetic vitreous samples. Although the direct involvement of any of these proteins in retinal injury and inflammation has not yet been investigated, pathways involving some of these proteins are implicated in various cellular processes, including membrane integrity, signal transduction, myelination, and calcium homeostasis, which are crucial in the pathophysiology of DR [29,30,31,32,33,34]. These findings are also consistent with existing literature that highlights inflammation, immune response, and metabolic dysregulation as key factors in DR progression. For instance, the upregulation of ITPR2 and CHERP suggests altered calcium signaling and homeostasis, which have been implicated in retinal cell death and vascular dysfunction in DR [12]. Similarly, increased levels of OMG and CORO1A align with previous reports of neuroinflammatory responses and cytoskeletal reorganization in DR [29]. The identification of these differentially expressed proteins may provide as valuable biomarkers for DR and potential targets for therapeutic interventions. Future studies focusing on validating these findings in larger, more diverse cohorts and exploring the functional roles of these proteins in DR pathogenesis is warranted.

Conversely, proteins such as Immunoglobulin heavy variable 3-64D, mRNA cap guanine-N7 methyltransferase, Adhesion G protein-coupled receptor B3, Kinesin-like protein KIF1B, Aldehyde dehydrogenase (mitochondrial), Dipeptidyl peptidase 4, Triokinase/FMN cyclase, G-protein-signaling modulator 1, Protein argonaute-1, and Plexin-B3 were expressed at lower levels in diabetic vitreous. Some of these downregulated proteins are associated with pathways that regulate the immune response, mRNA processing, cell adhesion, intracellular transport, mitochondrial dysregulation, and metabolic regulation [25,35,36], indicating potential disruptions in these pathways in DR.

Our pathway enrichment analysis of significantly altered proteins in the vitreous humor of diabetic individuals has revealed key insights into the molecular mechanisms underlying DR. Using databases such as Panther, KEGG, Reactome, and BioCarta, we identified 26 dysregulated pathways within the diabetic vitreous. The top KEGG pathways implicated include glycerolipid metabolism, pantothenate and CoA biosynthesis, histidine metabolism, and ascorbate and aldarate metabolism, highlighting the profound metabolic alterations in DR. These findings align with previous studies that have reported metabolic dysregulation as a hallmark of DR pathogenesis [19,29].

In the Panther database, several key signaling pathways were identified, such as Wnt signaling, heterotrimeric G-protein (Gαq and Gαo)-mediated pathways, chemokine and cytokine-mediated inflammation, and histamine H1 receptor-mediated signaling. These pathways are crucial for cellular communication and inflammatory responses, which are known to be also disrupted in DR [30]. The involvement of inflammatory signaling pathways underscores the role of chronic inflammation in retinal damage and progression of DR, consistent with previous literature that highlights inflammation as a central component in DR [12]. BioCarta pathway analysis, although revealing fewer altered pathways, identified critical processes such as the cycling of Ran in nucleocytoplasmic transport [37,38,39] and the role of PI3K subunit p85 in regulating actin organization and cell migration [40]. Therefore, these pathways are pertinent to retinal pathogenesis as they are involved in maintaining cellular structure and facilitating cellular responses to stress. The disruption of these pathways could lead to cytoskeletal abnormalities and impaired cellular migration, contributing to the retinal alterations observed in DR. Reactome enrichment analysis further supported the involvement of several regulatory pathways, including transcriptional regulation by MECP2, Ca^2+^ signaling, regulation by small RNAs, fructose catabolism, and MET/PTPN11 and MET/TNS protein signaling. The dysregulation of these pathways suggests a complex interplay between genetic regulation, calcium homeostasis, and metabolic pathways in the progression of DR [41,42]. The significant alteration in transcriptional regulation and calcium signaling aligns with the findings of previous studies that have linked these processes to neurodegeneration and vascular dysfunction, including retinal [43,44,45,46]. Overall, these results provide a comprehensive overview of the dysregulated pathways in diabetic vitreous humor, reinforcing the multifactorial nature of DR. These findings, however, highlight the need for further research to validate these pathways and explore their potential as therapeutic targets.

Our study comes with several limitations. First, the sample size is relatively small, preventing a full analysis of the patient characteristics, including the identification of the differences in risk factors associated with patients. Thus, a larger sample size is needed to detect such significant differences. Second, the vitreous samples were collected postmortem, and some of the patients had other diseases, such as heart disorders, which could have influenced the gene ontology analysis, such as molecular functions and biological processes, leading to potentially irrelevant findings regarding eye diseases. Third, though some of the patients had around 20 years of being diabetic, the development of DR is not confirmed (or data are unavailable) but is expected to occur based on the duration of diabetes. Fourth, the samples were taken from only type 2 diabetic patients; thus, type 1 diabetes was not feasible in our analysis. Finally, the exact role of each differentially expressed protein that is either highly or lower expressed in the context of diabetes deserves further experimental consideration, depending on the significant change levels and contributions to the pathogenesis of eye diseases.

## 5. Conclusions

Our study sheds light on the intricate molecular mechanisms underlying a leading cause of vision impairment globally. Through proteomic analysis of diabetic vitreous humor, we unveiled significant alterations in protein expression profiles associated with DR pathogenesis. In summary, our findings align with previous research, highlighting the critical roles of inflammation, immune response dysregulation, and metabolic disturbances driving DR. Specifically, the upregulated proteins point to aberrant calcium signaling, inflammatory responses, and cytoskeletal reorganization as key factors in DR. Conversely, the downregulated proteins suggest disruptions in immune function and cell adhesion processes. Our pathway enrichment analysis revealed several dysregulated pathways, including those involved in glycerolipid metabolism, inflammatory signaling, and transcriptional regulation, underscoring the multifactorial nature of DR. These insights deepen our understanding of the complex mechanisms underlying DR and may inform the development of targeted therapeutic strategies. Integrating proteomic data with other omics approaches, such as lipidomics and metabolomics, will further enhance our understanding of DR and facilitate the development of comprehensive treatment strategies [18,35]. By integrating our findings with existing literature, we pave the way for further research aimed at elucidating the complex pathogenesis of DR and translating these insights into clinical practice.

## Figures and Tables

**Table 1:** Demographics of the type 2 diabetic patients and non-diabetic controls.

	Diabetic	Non-Diabetic	*p* Value
n	6	6	
Age (years)	72 ± 12.17	79.83 ± 11.34	NS
Race			
Caucasian	5 (83.3)	6 (100)	NS
Black	1 (16.7)	0 (0)	NS
Gender, n (%)			
Male	3 (50)	3 (50)	NS
Female	3 (50)	3 (50)	NS
BMI (kg/m^2^)	33 ± 6.977	29.39 ± 5.214	NS
Weight (lbs)	187.9 ± 55.4	180.1 ± 46.7	NS
Height (inches)	66.55 ± 5.735	65.25 ± 4.26	NS
Tobacco use, n (%)	4 (66.7)	5 (83.3)	NS
Alcohol use, n (%)	3 (50)	3 (50)	NS
Duration of diabetes (years)	14.33 ± 9.933	0	<0.001
Complications, n (%)			
DR Status	Unknown	Unknown	NA
Heart attack/failure	5 (83.3)	4 (66.7)	NS
Medication, n (%)			
Hypoglycemic medications	6 (100)	0 (0)	<0.0001
Heart medications	5 (83.3)	5 (83.3)	NS

Data are shown as mean ± SD. *p*-value, diabetic versus nondiabetic controls. Abbreviations: BMI, body mass index; NS, not significant; NA, Not Applicable; SD, standard deviation.

**Table 2:** Ten most abundant proteins present in human vitreous samples.

#	Accession Number	Gene Symbol	Gene Name	Σ# PSMs		PSM Difference	Molecular Weight [kDa]
				Diabetic	Normal		
1	P02768	*ALB*	Albumin	39,462	35,582	3880	69.3
2	P01834	*IGKC*	Immunoglobulin kappa constant	1601	1723	−122	11.8
3	P02787	*TRFE*	Serotransferrin	7676	7902	−226	77.0
4	P01024	*C3*	Complement	1869	1542	327	187.0
5	P01009	*α1AT*	Alpha-1-antitrypsin	3039	2660	379	46.7
6	P01859	*IGHG2*	Immunoglobulin heavy constant gamma 2	1558	1522	36	35.9
7	P02790	*HPX*	Hemopexin	1440	1156	284	51.6
8	P0DOX5	*IGHG1*	Immunoglobulin gamma-1 heavy chain	2685	2343	342	49.3
9	P02763	*α1AG1*	Alpha-1-acid glycoprotein	1739	1504	235	23.5
10	P01860	*IGHG3*	Immunoglobulin heavy constant gamma 3	1579	1604	−25	41.3

**Table 3:** Proteins significantly higher in diabetic human vitreous samples.

#	Accession #	Protein	Symbol	*p* Value
1	A6NFY4	Nuclear envelope integral membrane protein 2	*NEMP2*	0.031067
2	B7Z6K7	Zinc finger protein 814	*ZNF814*	0.049418
3	P23515	Oligodendrocyte-myelin glycoprotein	*OMG*	0.041425
4	P31146	Coronin-1A	*CORO1A*	0.042233
5	Q14571	Inositol 1,4,5-trisphosphate receptor type 2	*ITPR2*	0.018265
6	Q6V1P9	Protocadherin-23	*DCHS2*	0.031779
7	Q8IV33	Uncharacterized protein KIAA0825	*KIAA0825*	0.030202
8	Q8IWX8	Calcium homeostasis endoplasmic reticulum protein	*CHERP*	0.019858
9	Q9UKX2	Myosin-2	*MYH2*	0.048992
10	Q9Y2H8	Zinc finger protein 510	*ZNF510*	0.036702

**Table 4:** Proteins significantly lower in diabetic human vitreous samples.

#	Accession #	Protein	Symbol	*p* Value
1	A0A0J9YX35	Immunoglobulin heavy variable 3-64D	*IGHV3-64D*	0.043559
2	O43148	mRNA cap guanine-N7 methyltransferase	*RNMT*	0.020019
3	O60242	Adhesion G protein-coupled receptor B3	*ADGRB3*	0.015424
4	O60333	Kinesin-like protein KIF1B	*KIF1B*	0.045349
5	P05091	Aldehyde dehydrogenase, mitochondrial	*ALDH2*	0.002538
6	P27487	Dipeptidyl peptidase 4	*DPP4*	0.049516
7	Q3LXA3	Triokinase/FMN cyclase	*TKFC*	0.034098
8	Q86YR5	G-protein-signaling modulator 1	*GPSM1*	0.048014
9	Q9UL18	Protein argonaute-1	*AGO1*	0.033237
10	Q9ULL4	Plexin-B3	*PLXNB3*	0.041133

**Table 5:** Reactome pathways significantly modulated in diabetic vitreous.

Index	Name	*p* Value
1	Infectious disease	0.000995
2	Transcriptional regulation By MECP2	0.004668
3	Ca^2+^ pathway	0.004821
4	Semaphorin interactions	0.005294
5	RUNX1 regulates genes involved in megakaryocyte differentiation and platelet function	0.005621
6	Transcriptional regulation by small RNAs	0.007389
7	Processing of capped intron-containing pre-mRNA	0.007940
8	Fructose catabolism	0.008472
9	MET activates PTPN11	0.008472
10	MET interacts with TNS proteins	0.008472

**Table 6:** Human phenotype ontology associated with altered proteins in diabetic vitreous.

Index	Name	*p*-Value
1	Pheochromocytoma	0.01352
2	Neuroendocrine neoplasm	0.01520
3	Congenital sensorineural hearing impairment	0.01687
4	Muscle fiber inclusion bodies	0.01855
5	Cerebral hemorrhage	0.02188
6	Neoplasm of the peripheral nervous system	0.02521
7	Bony spicule pigmentary retinopathy	0.03182
8	Abnormality of dental color	0.03347
9	Attenuation of retinal blood vessels	0.03512
10	Steppage gait	0.03840

## Data Availability

All the data has been included in the manuscript and the raw data is included in the Supplemental File.

## Supplementary Materials

The following supporting information can be downloaded at: https://www.mdpi.com/article/10.3390/life14070883/s1, Table S1: Multi-consensus proteomic analysis of vitreous samples.
